# In Situ Fabrication of Bi_2_Ti_2_O_7_/TiO_2_ Heterostructure Submicron Fibers for Enhanced Photocatalytic Activity

**DOI:** 10.1186/s11671-016-1408-7

**Published:** 2016-04-12

**Authors:** Di Zhou, Hu Yang, Yafang Tu, Yu Tian, Yaxuan Cai, Zhenglong Hu, Xiaolong Zhu

**Affiliations:** School of Physics and Information Engineering, Jianghan University, Wuhan, 430056 China; Faculty of Physics and Electronic Technology, Hubei University, Wuhan, 430062 China; Laboratory of Low-Dimension Functional Nanostructures and Devices, Hubei University of Science and Technology, Xianning, 437100 China

**Keywords:** Bismuth titanate, Heterostructure, Hydrothermal, Visible light, Photocatalyst

## Abstract

A facile two-step synthesis route combining electrospinning and hydrothermal techniques has been performed to obtain Bi_2_Ti_2_O_7_/TiO_2_ heterostructured submicron fibers. Bi_2_Ti_2_O_7_ nanosheets were grown on the surface of TiO_2_ submicron fibers. The density of the nanosheets increased with higher precursor concentration of the Bi/Ti reaction raw materials. UV-visible (UV-vis) diffuse reflectance spectroscopy indicated that the absorption spectrum of the Bi_2_Ti_2_O_7_/TiO_2_ composite extended into the visible-light region. Photocatalytic tests showed that the Bi_2_Ti_2_O_7_/TiO_2_ heterostructures possess a much higher degradation rate of rhodamine B than the unmodified TiO_2_ submicron fibers under visible light. The enhanced photocatalytic activity can be attributed to the synergistic effect between improved visible-light absorption and the internal electric field created by the heterojunctions. The effective separation of photogenerated carriers driven by the photoinduced potential was demonstrated by the photoelectrochemical analysis.

**Electronic supplementary material:**

The online version of this article (doi:10.1186/s11671-016-1408-7) contains supplementary material, which is available to authorized users.

## Background

Over the past decades, a large number of metal oxides have been explored for the purpose of photocatalytic degradation of harmful organic substances and hydrogen production through splitting water. Titanium dioxide (TiO_2_) is widely regarded as a heterogeneous photocatalyst for the photodegradation of pollutants in wastewater due to its low-cost, strong oxidizing power, non-toxicity, and long-term photostability [1–4]. For certain practical applications, however, pure TiO_2_ is not very suitable because it only absorbs UV light at wavelengths no longer than 387.5 nm (anatase phase) or 413.3 nm (rutile phase). Even complete absorption in that range would account for less than 5 % of incoming solar light energy. Additionally, the high recombination rate of photogenerated electron-hole pairs is another problem if TiO_2_ is used for photocatalysis [4]. A common method used to overcome these drawbacks is to create a heterojunction composite comprising a TiO_2_ bottom-layer and a different top-layer semiconductor with a narrow band gap. That way, the built-in potential gradient at the interface between the semiconductors facilitates the separation of electron-hole pairs and reduces the chance of recombination [5–8]. For effective sensitization of TiO_2_ using another semiconductor to capture a larger part of the solar spectrum, the second semiconductor should meet the following conditions [5, 6]: (1) be a narrow band gap semiconductor; (2) possess a lower anodic conduction band (CB) than TiO_2_; (3) possess high stability to prevent photocorrosion; and (4) be a visible light-driven photocatalyst itself. Thus, the development of suitable methods to fabricate narrow band gap semiconductor/TiO_2_ heterojunction composites will be essential for the practical application of TiO_2_ as photocatalyst.

Bismuth titanates, a large family that includes several phases of the Bi-O-Ti system, including Bi_2_Ti_4_O_11_, Bi_2_Ti_2_O_7_, Bi_4_Ti_3_O_12_, Bi_20_TiO_32_, and Bi_12_TiO_20_, are promising candidates for many technological applications [9–14]. Several of these materials have been reported as visible light-driven photocatalysts, for example: Bi_2_Ti_2_O_7_ nanorods, Bi_12_TiO_20_ nanowires, and Bi_20_TiO_32_ nanosheets [12–14]. Especially, Bi_2_Ti_2_O_7_ with its pyrochlore structure forms a shallow acceptor energy level in the forbidden band because of a non-stoichiometric ratio of Bi_1.74_Ti_2_O_6.62_ and the Bi vacancy [15]. As a result, the holes in Bi_2_Ti_2_O_7_ can be excited into the valence band (VB) under irradiation with visible light. Bian and Ren et al. reported independently that Bi_2_Ti_2_O_7_ absorbs well in the visible region and also possesses enhanced photocatalytic activity with regard to the decomposition of rhodamine B (RhB) under visible light [16, 17]. However, the fast recombination of photogenerated electron-hole pairs seriously limits energy-conversion efficiency. To promote the separation of photogenerated carriers in Bi_2_Ti_2_O_7_, designing a composite photocatalyst by coupling Bi_2_Ti_2_O_7_ with a semiconductor with matched band potentials is a sensible strategy. For example, Wang and Hou et al. reported independently that Bi_2_Ti_2_O_7_/TiO_2_ composite powders and nanowire arrays showed higher photocatalytic activity than pure TiO_2_ under visible light [18–20]. Based on the above considerations, the authors constructed a novel Bi_2_Ti_2_O_7_ sensitized TiO_2_ composite system, with the goal to expand the photocatalytic activity of TiO_2_ into the visible-light range. However, the photocatalytic performance of common composite films with a dense and smooth surface is still moderate. This is because the redox reaction occurs on the surface of catalyst film, and the surface morphology of the catalyst film plays a key role in the photocatalytic property [20–22]. Therefore, Bi_2_Ti_2_O_7_ nanosheets-TiO_2_ submicron fibers heterostructures were chosen as tested candidates and fabricated by using a combination of an electrospinning technique and hydrothermal method.

In this work, a facile in situ hydrothermal method was used to grow secondary Bi_2_Ti_2_O_7_ nanostructures on TiO_2_ submicron fibers. Electrospun TiO_2_ submicron fibers were employed because the fiber matrix possesses the favorable morphology of high surface areas and aspect ratios [23, 24]. Moreover, it can serve as both reactant and substrate, ensuring close contact between Bi_2_Ti_2_O_7_ nanostructures and TiO_2_ submicron fibers for uniform growth of a hierarchical configuration. The hydrothermal process was performed in an alkaline environment where an aqueous solution of Bi(NO_3_)_3_ and submicron TiO_2_ fibers were used as reactants [25–27]. The characterization results indicated that Bi_2_Ti_2_O_7_ nanosheets with high crystallinity grew successfully on TiO_2_ submicron fibers and well-defined three-dimensional hierarchical heterostructures of Bi_2_Ti_2_O_7_/TiO_2_ submicron fibers were formed. In contrast to pure TiO_2_ and Bi_2_Ti_2_O_7_, the composites showed significantly improved light absorption at a wavelength above 420 nm, as well as higher photocurrent density under a visible-light pulse. Photocatalytic tests revealed that the Bi_2_Ti_2_O_7_/TiO_2_ heterostructures have higher visible-light activity for degrading RhB than the pure Bi_2_Ti_2_O_7_, and unmodified TiO_2_.

## Methods

TiO_2_ submicron fibers were fabricated using the well-known process reported previously in Refs. [20, 23]. In the following hydrothermal procedure, 5 mg of the electrospun TiO_2_ submicron fibers were placed into an autoclave containing two different Bi(NO_3_)_3_ solutions. The concentration of the Bi(NO_3_)_3_ solutions was 0.0103 and 0.0412 mmol L^−1^, respectively. The pH value of the solution was adjusted to 13 using a 1 M KOH solution. The reaction was carried out at 180 °C for 24 h. The fabricated products were collected, washed with deionized water, and then dried in an oven at 60 °C for 6 h. Using this method, two different Bi_2_Ti_2_O_7_/TiO_2_ composites were produced, which were denoted as BT1 and BT2, respectively.

The structure and morphology of the prepared samples was investigated using powder X-ray diffraction (XRD; Bruker D8 Advance, using Cu*K*α radiation), scanning electron microscopy (SEM; Hitachi S-4800), and transmission electron microscopy (TEM; JEOL 2100). The optical properties of the samples were analyzed via UV-visible diffuse reflectance spectroscopy, recorded on a UV/Vis spectrophotometer (Shimadzu UV-2550) at room temperature. Photoelectrochemical measurements of the prepared samples were recorded with a laboratory-built electrochemical analyzer (CHI660E, China) consisting of a standard three-electrode system [20]. The Bi_2_Ti_2_O_7_/TiO_2_ heterojunction composite films served as working electrodes after coating the produced samples on Au/SiO_2_/Si substrates (10 × 10 mm). A 300-W Xe lamp, equipped with a 420 nm cutoff filter was used for excitation source.

To measure photocatalytic activity, a 100 ml of rhodamine B (RhB; 1.0 × 10^−5^mol L^−1^) solution with an initial concentration of 10 mg L^−1^ in the presence of solid catalyst was filled into a laboratory-built photoreactor. The photoreactor was equipped with an internal light source (150-W Xe lamp and a cutoff filter transmitting >420 nm) surrounded by a water-cooled quartz barrier to cool the lamp. The solution with the photocatalysts was stirred in the dark for 30 min to obtain a good dispersion and establish an adsorption-desorption equilibrium between the organic molecules and the catalyst surface. Changes in concentration of the dye solution were measured with a spectrophotometer at lambda of 553 nm at specified reaction intervals.

## Results and Discussion

**Fig. 1:**
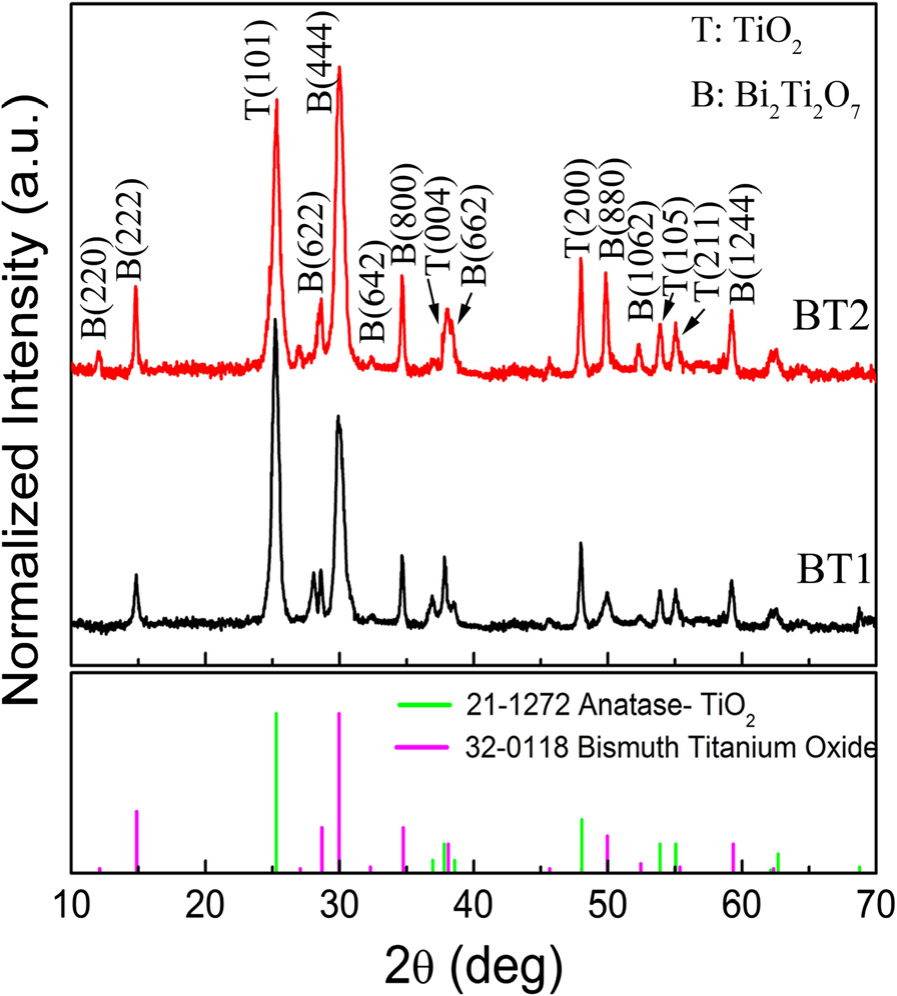
XRD patterns of typical Bi_2_Ti_2_O_7_/TiO_2_ composites

The crystal structures of Bi_2_Ti_2_O_7_/TiO_2_ composites were identified via XRD analysis, as shown in Fig. 1. The strong, sharp peaks indicated that the as-obtained products are highly crystallized. For the sample BT1, diffraction peaks at about 2*θ* = 25.1°, 37.4°, 48.2°, 54.1°, and 55.0° could be indexed perfectly to the (101), (004), (200), (105), and (211) crystal planes of anatase TiO_2_ (JCPDS 21-1272,), respectively. Additional diffraction peaks appear with 2*θ* values of 14.9°, 28.8°, 30.2°, 32.1°, 34.6°, 37.8°, 49.6°, and 52.3°, which correspond to (222), (622), (444), (642), (800), (662), (880), and (10, 6, 2) crystal planes of the cubic phase of Bi_2_Ti_2_O_7_, respectively (JCPDS 32-0118,). This suggests that part of TiO_2_ has been successfully converted to Bi_2_Ti_2_O_7_. For the sample BT2, the relative intensity of diffraction peaks for the ratio of BT2 to TiO_2_ became stronger than that of BT1 to TiO_2_. This suggests a higher yield of Bi_2_Ti_2_O_7_ in sample BT2, which was further confirmed after observation with the SEM. Additionally, no impurity-attributed peaks were detected in the patterns of the XRD analysis. The XRD peaks of TiO_2_ in the two Bi_2_Ti_2_O_7_/TiO_2_ composites did not shift compared with the standard diffractive peaks of pure anatase TiO_2_, which indicates that Bi did not substitute Ti and enter the TiO_2_ lattices. Therefore, it appears that the synthesis route was favorable for obtaining a multicomponent oxide composite that integrates the anatase phase of TiO_2_ with Bi_2_Ti_2_O_7_.

**Fig. 2:**
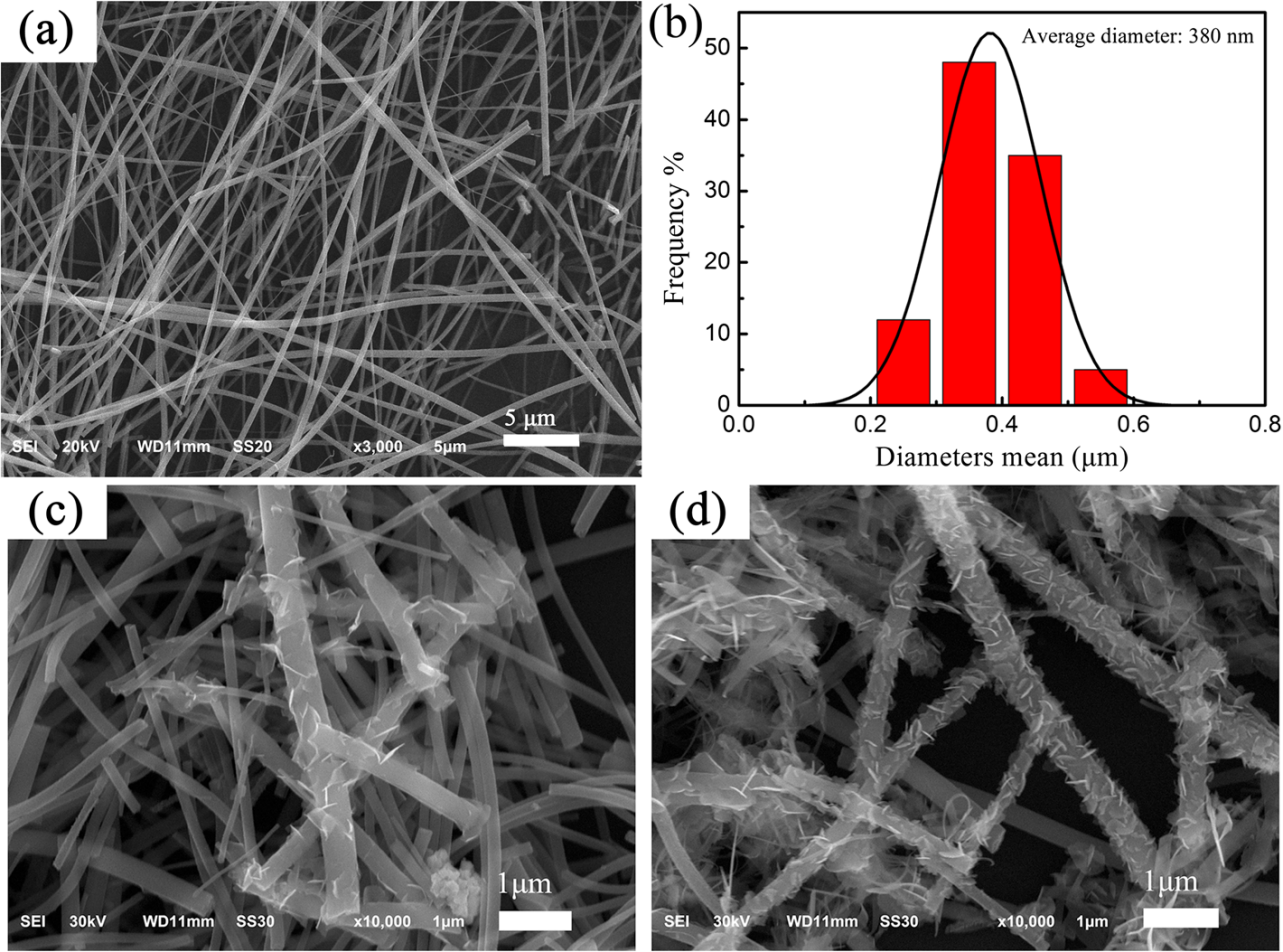
**a** SEM images of TiO_2_ submicron fibers and **b** the corresponding histogram of diameters distribution. **c**, **d** SEM images of the composition BT1 and BT2, respectively

Figure 2 shows the morphologies and distribution of the mean diameters of the fibered samples. Before hydrothermal treatment, the TiO_2_ submicron fibers with diameters about 200–600 nm had a relatively smooth surface without secondary nanostructures, and the average diameter was estimated to be about 380 nm, as shown in Fig. 2a, b. After the hydrothermal treatment, the samples remained as a non-woven fibrous morphology. However, the surface was no longer relatively smooth. Instead, the submicron fibers were decorated with numerous secondary nanosheets, as shown in Fig. 2c, d. After increasing the concentration of the Bi(NO_3_)_3_ precursor by a factor four, the density of the nanosheets grown on the surface of submicron fibers increased significantly. This result is confirmed by the XRD analysis. The size of the nanosheets, however, showed no obvious changes. The nanosheets were still uniformly distributed along each fiber—without aggregation—although their density increased significantly. This might be because the high porosity and large surface area of the TiO_2_ fibers favor both growth and uniform distribution of secondary nanostructures.

**Fig. 3:**
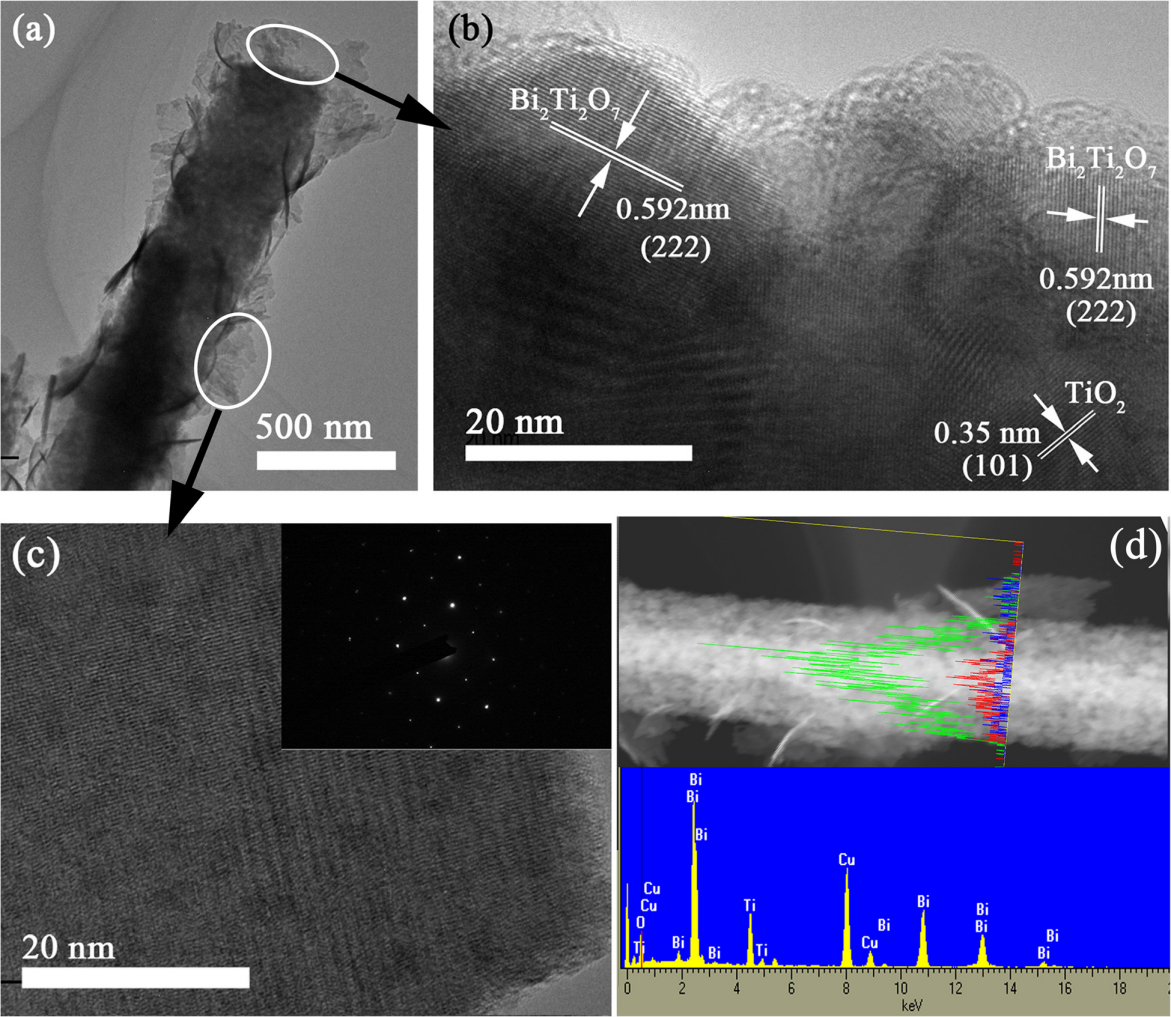
**a** TEM image of Bi_2_Ti_2_O_7_/TiO_2_ composite fiber. **b** HRTEM image of heterojunction region. **c** HRTEM image of the nanosheet surface. The *inset* shows SAED of the single nanosheet. **d** Elemental line mapping along a radial direction of the Bi_2_Ti_2_O_7_/TiO_2_ fiber showing Bi (blue), Ti (red), and O (green) and EDX spectrum

The typical TEM images of an individual hierarchical submicron fiber of sample BT2 were shown in Fig. 3. The Bi_2_Ti_2_O_7_ that grew around the TiO_2_ matrix shows a nanosheet configuration with a narrow and uniform size distribution, which is consistent with the SEM images in Fig. 3. The HRTEM image of the junction clearly showed two types of lattice fringes, as shown in Fig. 3b. For one set of fringes, the spacing is 0.35 nm, which corresponds to the (101) plane of the anatase crystal structure of TiO_2_. For the other set of fringes, the spacing is 0.592 nm, which corresponds to the (111) lattice spacing of cubic Bi_2_Ti_2_O_7_. The results suggest the presence of heterojunctions, which can improve both charge separation and charge transfer within the hybrid structure over pure Bi_2_Ti_2_O_7_ and TiO_2_. A selected area electron diffraction (SAED) pattern was characterized from a single nanosheet (Fig. 3c). The bright diffraction spots clearly reveal the high crystallinity of a single crystal Bi_2_Ti_2_O_7_ nanosheet. Figure 3d shows elemental line mapping of Bi, Ti, and O concentrations along a radial direction of the Bi_2_Ti_2_O_7_/TiO_2_ composite fibers. All elements (Bi, Ti, and O) were observed to be homogeneously distributed in the fibers. Further, the quantitative analysis of the products was measured by using X-ray fluorescence (XRF) spectrum. As shown in Additional file 1: Figure S1, the molar ratio of Bi_2_Ti_2_O_7_ to TiO_2_ could be determined as 1/14 for BT1 and 1/3.5 for BT2, respectively. (Detailed derivation process is described in the supporting information.)

**Fig. 4:**
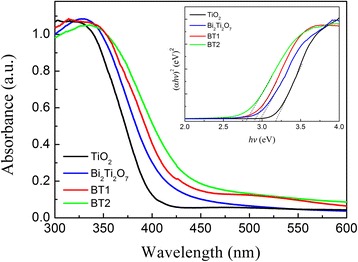
UV-visible absorption spectra and (*inset*) the relationship between (*αhv*)^2^ and band gap energy (*E*_*g*_) for different samples

Figure 4 shows the UV-Vis absorption spectra of the composites BT1 and BT2, as well as pure phase TiO_2_ submicron fibers and the Bi_2_Ti_2_O_7_ hydrothermal product, which were converted from the corresponding diffusion reflectance spectra using the Kubelka-Munk function [27]. Pure anatase TiO_2_ submicron fibers only showed fundamental absorption in the UV-light region, while Bi_2_Ti_2_O_7_ displayed a portion of absorption in visible-light region. For the heterostructures, the curves of samples BT1 and BT2 showed a shift of the absorption edge toward longer wavelengths, i.e., into the visible-light region. The absorption of visible light showed a progressive red shift with the increasing density of the Bi_2_Ti_2_O_7_ nanosheets grew on TiO_2_ submicron fibers. This suggests a potential ability for photocatalytic decomposition of organic contaminants under irradiation with visible light. For semiconductors of the direct transition type, the relation curves of (*αhv*)^2^ versus band gap energy *E*_*g*_ were obtained using the equation (where *α* is absorption coefficient) [28, 29]: *hv* − *E*_*g*_ = (*αhv*)^2^. Therefore, their respective band gaps were calculated as 3.2, 2.94, 2.86, and 2.78 eV for pure TiO_2_, Bi_2_Ti_2_O_7_ and the heterostructured composites BT1 and BT2. The reduced band gap energy for heterostructures can be attributed to the formation of an internal electric field between Bi_2_Ti_2_O_7_ and TiO_2_, which causes the Fermi levels of the two materials to move toward each other and eventually reach the same potential. The improved light absorption of the composites creates more electron-hole pairs for unchanged visible-light irradiation, which subsequently results the potential of enhanced photocatalytic activity.

**Fig. 5:**
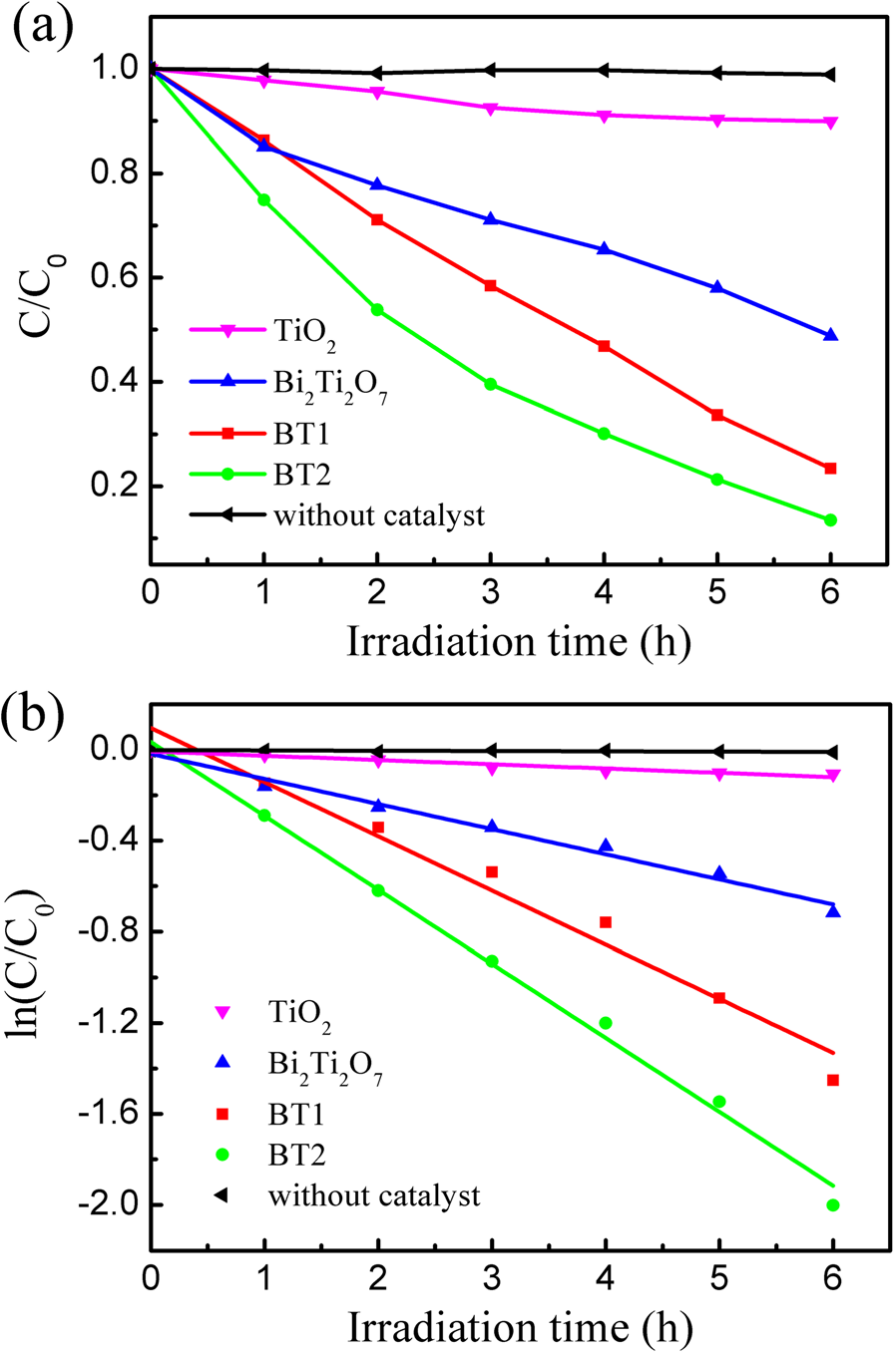
**a** Degradation curves of RhB under visible light. **b** Reaction rate correlation for RhB degradation on different catalysts

The photocatalytic degradation of RhB had been chosen as a model reaction to evaluate the photocatalytic activity of the present Bi_2_Ti_2_O_7_/TiO_2_ heterostructure. Adsorption in the dark was performed to ensure sufficient dispersion and an adsorption-desorption equilibrium between the organic molecules and the catalyst surface. Figure. 5a shows a series of plots of the RhB degradation efficiency (*C*/*C*_0_) of four different photocatalysts: pure phase TiO_2_ submicron fibers, Bi_2_Ti_2_O_7_ hydrothermal product, and Bi_2_Ti_2_O_7_/TiO_2_ heterostructure submicron fibers (BT1 and BT2). The performance of RhB degradation without any catalyst also was carried out to be a comparison. *C*_0_ and *C* represent the RhB concentration at reaction time 0 and *t*, respectively. The order of photocatalytic activities under visible-light irradiation was manifested as: BT2 > BT1 > Bi_2_Ti_2_O_7_ > TiO_2_. Furthermore, the kinetic linear simulation curves of RhB photocatalytic degradation for the four different photocatalysts were shown in Fig. 5b. The degradation of RhB by composites under visible-light irradiation is known to follow the first-order Langmuire-Hinshelwood rate equation: *C* = *C*_0_ · *e*^− *kt*^, where *k* (min^−1^) denotes the pseudo-first-order rate constant of the reaction [8]. The rate constants (*k*) were calculated to be 3.13 × 10^−4^ min^−1^ for TiO_2_ and 1.84 × 10^−3^ min^−1^ for Bi_2_Ti_2_O_7_, as well as 3.97 × 10^−3^ min^−1^ for BT1 and 5.42 × 10^−3^ for BT2, respectively. For comparison, the photocatalytic performances of samples with different concentrations were shown in Additional file 1: Figure S2. The enhanced photocatalytic performance of the Bi_2_Ti_2_O_7_/TiO_2_ composite fibers was attributed to a synergistic effect between Bi_2_Ti_2_O_7_ and TiO_2_. Firstly, according to UV-Vis absorption analysis, the absorption edges of samples containing Bi_2_Ti_2_O_7_ shifted toward longer wavelengths and into the visible-light region, which increases the production of electron-hole pairs under the same visible-light irradiation. Secondly, compared with single Bi_2_Ti_2_O_7_ or TiO_2_, the presence of nanoscale heterojunctions in the composites promoted charge separation but also suppressed the recombination of already photogenerated electron-hole pairs [18, 30]. This is further confirmed by photoelectrochemical measurements mentioned below.

**Fig. 6:**
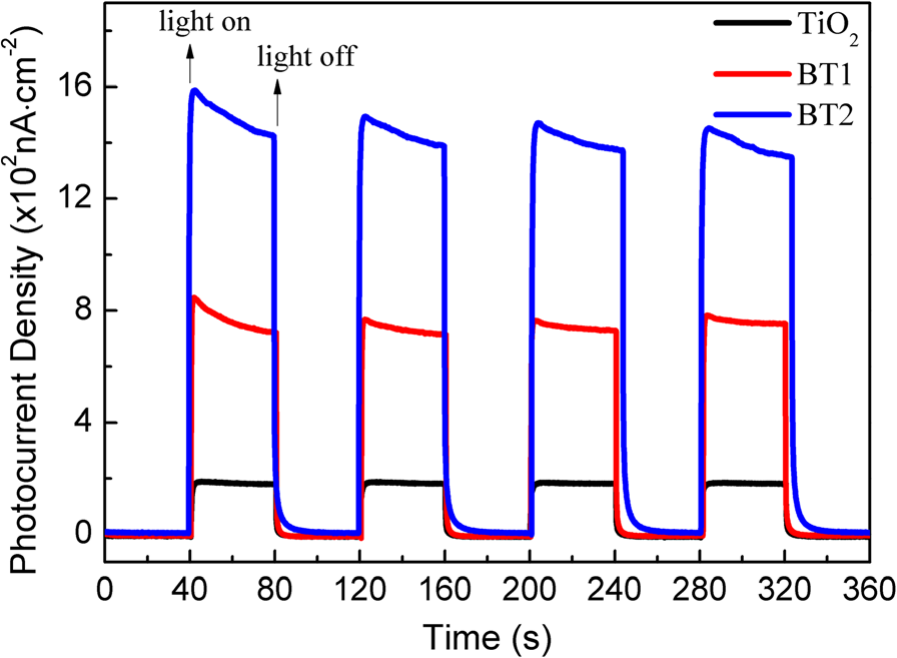
Photocurrent density versus time plotted for the different samples

The semiconducting nature of the Bi_2_Ti_2_O_7_/TiO_2_ composite fibers makes them suitable for the development of photoelectrochemical cells [28, 31]. In order to evaluate their relevant properties and further illustrate the enhanced electron transfer in the composites, the photocurrent − time (*I − t*) curves with several on-off cycles of intermittent visible-light irradiation were carried out, as shown in Fig. 6. The photocurrent responded immediately and rapidly increased to reach and maintain a constant value immediately after irradiation started. The photocurrent rapidly decreased and reached zero when the illumination stopped. This effect was reproduced and confirmed many times. The Bi_2_Ti_2_O_7_/TiO_2_ composite electrodes showed a strong instant photoresponse upon illumination with visible light, and provided a stable photocurrent values (0.72 μA cm^−2^ for BT1, and 1.44 μA cm^−2^ for BT2), which is considered to be superior to the TiO_2_ electrode (0.4 μA cm^−2^). The magnitude of the photocurrent is a measure of charge collection efficiency at the electrode surface. More electron-hole pairs were produced in BT2 with the greater density in hierarchical Bi_2_Ti_2_O_7_ compared to BT1, which results a higher photocurrent under the same visible-light irradiation. Moreover, the photoelectrochemical responses are identical, which confirms that the higher photocurrent solely originates from the coupling between Bi_2_Ti_2_O_7_ and TiO_2_.

**Fig. 7:**
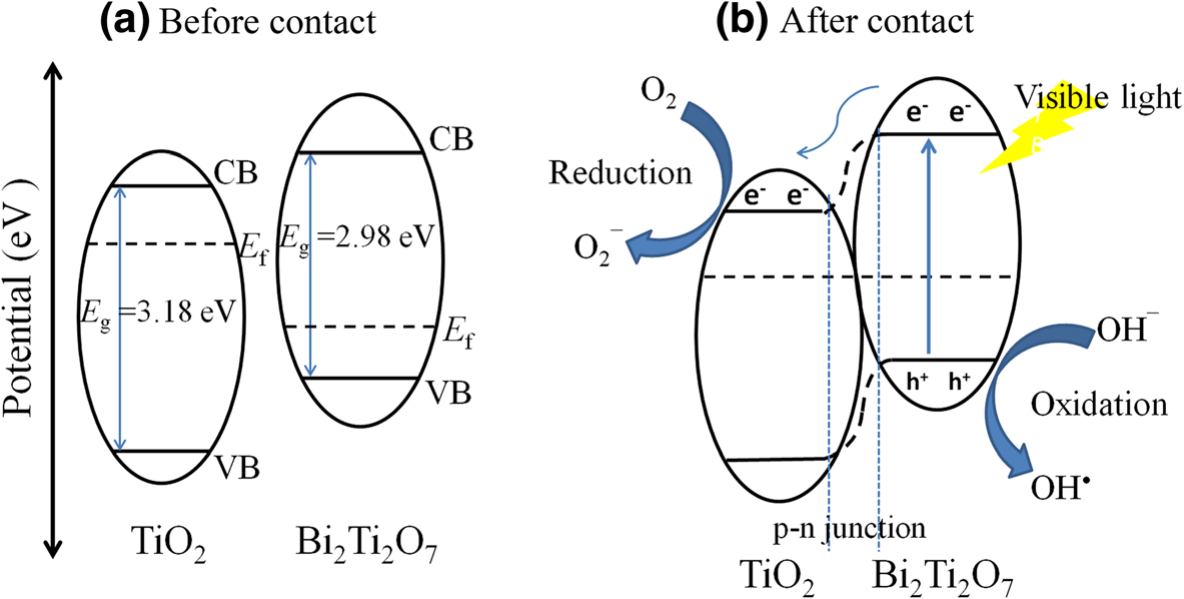
Schematic diagram of **a** the band energy of Bi_2_Ti_2_O_7_ and TiO_2_ before contact; **b** the formation of a p-n junction and the process of electron-hole pairs separation and transfer under visible-light irradiation

It is evident that the enhanced activity of the hybrid photocatalyst involving Bi_2_Ti_2_O_7_ and TiO_2_ can be attributed to the synergistic effects between visible light sensitization and the presence of heterojunctions. The CB and VB positions of semiconductor could be calculated using the empirical equation: *E*_CB_ = *X* − *E*_*e*_ − 0.5*E*_*g*_, where *X* is the geometric mean of Mulliken’s electron negativities of constituent atoms, *E*_e_ is the energy of free electrons on the hydrogen scale (about 4.5 eV), and *E*_g_ is the band gap energy [26]. When Bi_2_Ti_2_O_7_ is in contact with TiO_2_ to form a heterojunction, the Fermi levels of Bi_2_Ti_2_O_7_ and TiO_2_ tend to descend and ascend, respectively. This indicates that an inner electric field is created at the interface, followed by the formation of an equilibrium. It is known that photocatalytic processes are based on electron-hole pairs generated by means of band gap excitation. The generation and separation of the electron-hole pairs are the key factors to influence a photocatalytic reaction [30]. In this case, the RhB degradation over the Bi_2_Ti_2_O_7_/TiO_2_ composite under visible-light irradiation was carried out through several pathways, as illustrated in Fig. 7. The photogenerated electron-hole pairs were produced in Bi_2_Ti_2_O_7_ nanosheets after being excited with visible light with energies below 2.94 eV (*λ* > 420 nm). The generated electrons in the Bi_2_Ti_2_O_7_ nanosheets then migrated to the CB of TiO_2_, leaving holes in the VB of Bi_2_Ti_2_O_7_. As a result, the higher charge separation rate increased the lifetime of the charge carriers and enhanced the efficiency of the interfacial charges transferred to the adsorbed substrates, which in turn leads to higher activity of the Bi_2_Ti_2_O_7_/TiO_2_ composite photocatalyst. Meanwhile, the electrons (*e*^–^) generated in the CB react with dissolved oxygen molecules to produce superoxide radical anions ·O_2_^–^. The latter generates, via protonation, the hydroperoxy radicals ·HO_2_, which produce the hydroxyl radicals ·OH. The ·OH is a strong oxidizing agent to decompose the organic dye. The effect of reactive oxygen species (··OH and ·O_2_^–^) on the degradation of RhB was investigated by introducing isopropanol (IPA) and ammonium oxalate (AO) as the scavengers of ·OH and O_2_^–^, respectively. The quenching of the reaction containing scavengers indicated that ·OH is significant reactive species in the TiO_2_-catalyzed photocatalytic oxidation process [31, 32] (see Additional file 1: Figure S3 for photocatalytic performances of RhB aqueous solution containing scavengers). The reactions related to the degradation of RhB could be referred to the relevant literatures elsewhere [8, 18, 19, 31–34].

## Conclusions

In summary, a composite photocatalyst of Bi_2_Ti_2_O_7_/TiO_2_ heterostructured submicron fibers was synthesized via an in situ hydrothermal method. SEM and TEM observation revealed that the as-synthesized sample is micro-sized fiber-like hierarchy configuration consisted of Bi_2_Ti_2_O_7_ nanosheets decorated with the primary TiO_2_ submicron fibers. Extension of the light absorption from the ultraviolet region to the visible-light region was confirmed by UV-vis absorption spectra. The heterostructure of the Bi_2_Ti_2_O_7_/TiO_2_ composite exhibited enhanced visible photocatalytic activity over that of the pure Bi_2_Ti_2_O_7_ and TiO_2_ in the decomposition of RhB in water. The enhanced photocatalytic activity can be attributed to the extended absorption in the visible-light region and the effective separation of photogenerated carriers driven by the inner potential generated at the Bi_2_Ti_2_O_7_/TiO_2_ junction interface, which was demonstrated by the measurement of photocurrent response. Moreover, such a simple and versatile method could enable the use of TiO_2_ submicron fibers as precursor materials and templates to fabricate many other ternary titanate-based heterostructures for environmental applications and solar cell devices [35, 36].

## Additional file

Additional file 1: Figure S1. (a) and (b) The morphology images of the composition BT1 and BT2, respectively; (c) The XRF quantitative analysis of the molar ratio of Bi_2_Ti_2_O_7_/TiO_2_ in the corresponding composition BT1 and BT2. **Figure S2.** (a) Degradation curves and (b) reaction rate correlation of solid catalysts with different concentration in RhB aqueous solution. **Figure S3.** The effects of active species (·OH and ·O_2_^–^) on the degradation of RhB during the photocatalytic process. Detail derivation process for the molar ratio of Bi_2_Ti_2_O_7_ to TiO_2_. (DOC 2.82 mb)

## Abbreviations

BT1: Bi_2_Ti_2_O_7_/TiO_2_ composites with molar ratio of 1/14
BT2: Bi_2_Ti_2_O_7_/TiO_2_ composites with molar ratio of 1/3.5
CB: conduction band
JCPDS: Joint Committee on Powder Diffraction Standards
RhB: rhodamine B
SEM: scanning electron microscopy
TEM: transmission electron microscopy
UV: ultraviolet
UV-vis: UV-visible
VB: valence band
XRD: X-ray diffraction
