# Supplementary material for: In Situ Fabrication of Bi2Ti2O7/TiO2 Heterostructure Submicron Fibers for Enhanced Photocatalytic Activity
Source: Nanoscale Res Lett. 2016 Apr 12;11:193. doi: 10.1186/s11671-016-1408-7 (PMC4829568; doi:10.1186/s11671-016-1408-7)
Supplement: Additional file 1: Figure S1. — (a) and (b) The morphology images of the composition BT1 and BT2, respectively; (c) The XRF quantitative analysis of the molar ratio of Bi2Ti2O7/TiO2 in the corresponding composition BT1 and BT2. Figure S2. (a) Degradation curves and (b) reaction rate correlation of solid catalysts with different concentration in RhB aqueous solution. Figure S3. The effects of active species (·OH and ·O2 –) on the degradation of RhB during the photocatalytic process. Detail derivation process for the molar ratio of Bi2Ti2O7 to TiO2. (DOC 2.82 mb) [file 11671_2016_1408_MOESM1_ESM.doc]

**Supplementary Information**

***In situ* fabrication of Bi_2_Ti_2_O_7_/TiO_2_ heterostructure submicron fibers for enhanced photocatalytic activity**

Di Zhou ^1^, ⃰, Hu Yang ^1^, Yafang Tu ^1^, Yu Tian ^1^, Yaxuan Cai ^2^, Zhenglong Hu ^3^, Xiaolong Zhu ^1^

^1^ School of Physics & Information Engineering, Jianghan University, Wuhan 430056, China.

^2^ Faculty of Physics & Electronic Technology, Hubei University, Wuhan 430062, China.

^3^ Laboratory of Low-dimention functional Nanostructures and Devices, Hubei University of Science and Technology, Xianning 437100, China.

Correspondence: zhdijhu@gmail.com


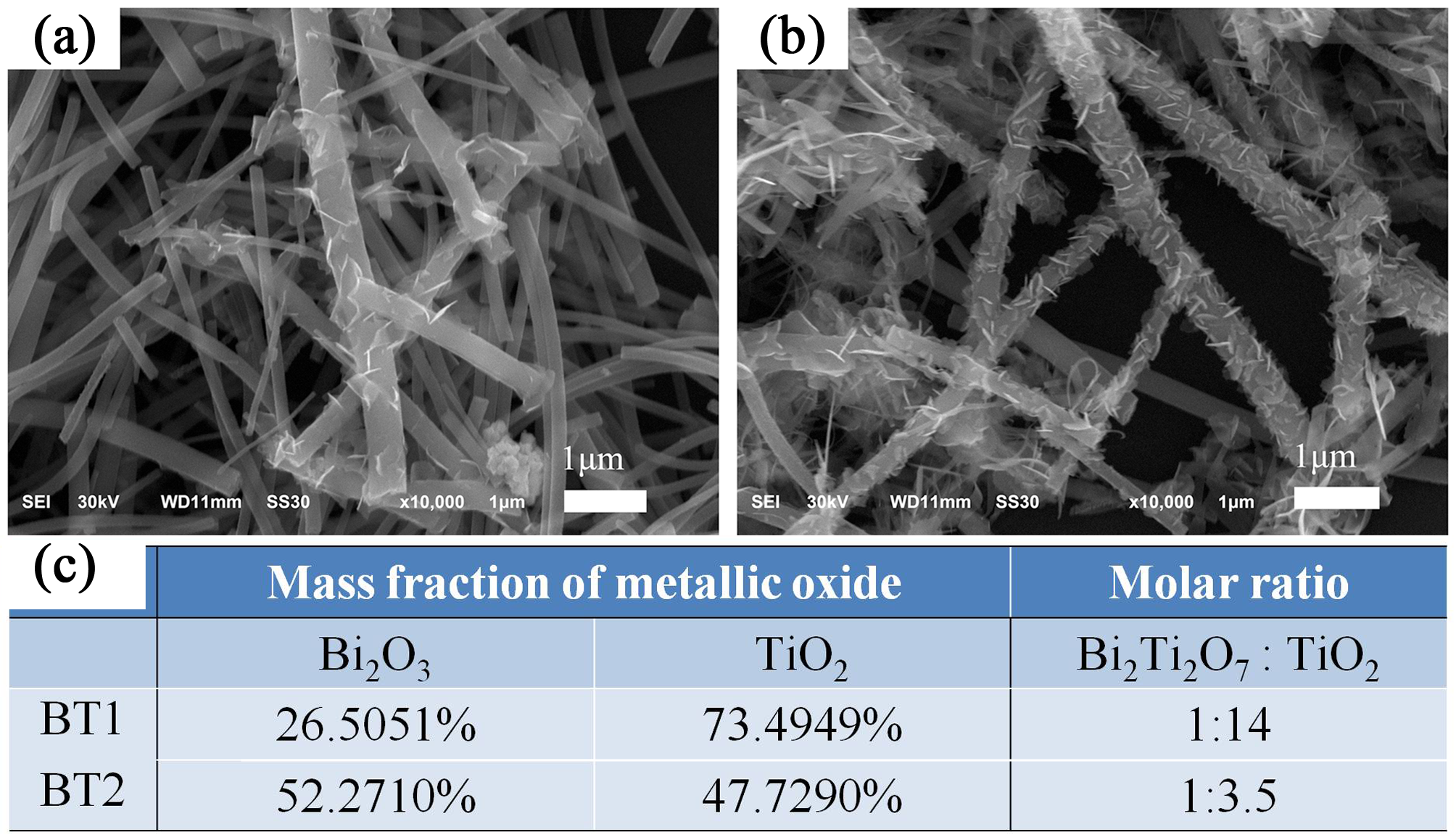


Figure S1: (a) and (b) The morphology images of the composition BT1 and BT2, respectively; (c) The XRF quantitative analysis of the molar ratio of Bi_2_Ti_2_O_7_/TiO_2_ in the corresponding composition BT1 and BT2.


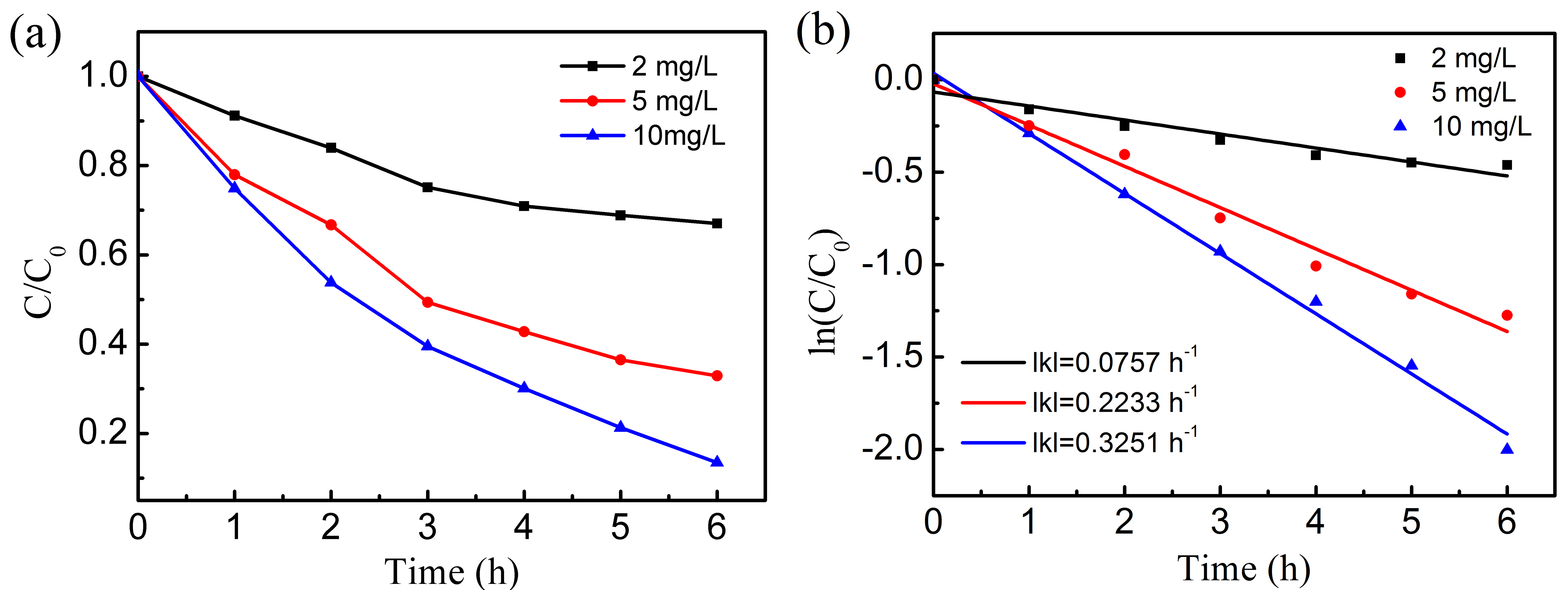


Figure S2: (a) Degradation curves and (b) reaction rate correlation of solid catalysts with different concentration in RhB aqueous solution.


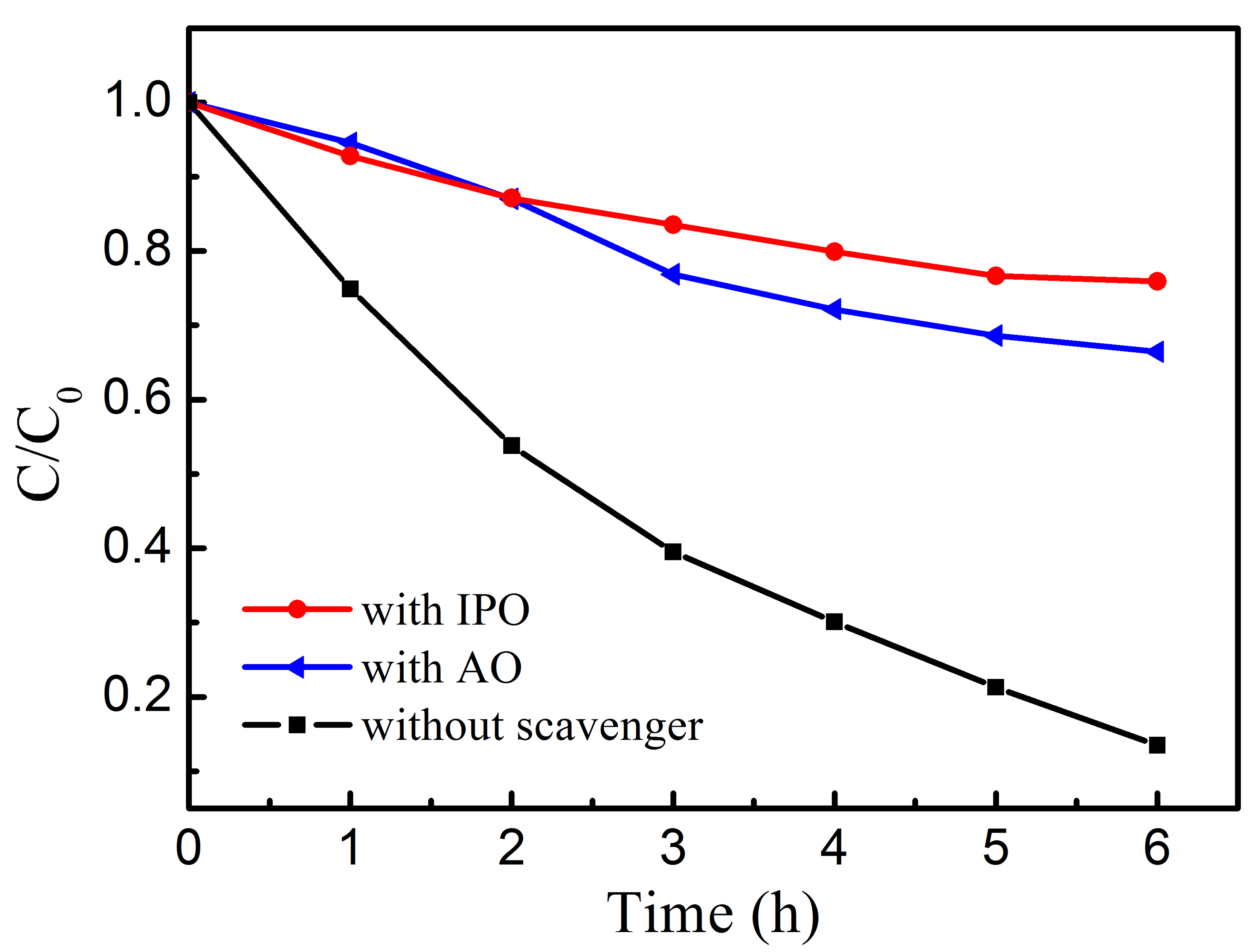


Figure S3: The effects of active species (•OH and •O_2_^−^) on the degradation of RhB during the photocatalytic process. The trapping experiments were carried out under the same conditions (10 mg/L of solid catalyst in 100 ml (1×10^-5^ M) of RhB aqueous solution, under visible-light irradiation), except for adding 1 mM of the scavengers. Isopropanol (IPA) and ammonium oxalate (AO) were used as scavenger of •OH and •O_2_^−^, respectively.

**Detail derivation process for the molar ratio of Bi_2_Ti_2_O_7_ to TiO_2_:** If the molar number of Bi_2_Ti_2_O_7_ and TiO_2_ is *a* and *b*, respectively, then the molar ratio of Bi_2_Ti_2_O_7_ to TiO_2_ (denoted as *R*) and the molar number of metallic oxide can be calculated as following: (where $m_{B}$, $m_{T}$ represent mass fraction and $\mu_{B}$, $\mu_{T}$ represent molar mass of Bi_2_O_3_ and TiO_2_, respectively)

$$R=\frac{a}{b}$$

$$a=\frac{m_{B}}{\mu_{B}}$$

$$2a+b=\frac{m_{T}}{\mu_{T}}$$
